# Molecular Structure and Phylogenetic Analyses of the Plastomes of Eight *Sorbus* Sensu Stricto Species

**DOI:** 10.3390/biom12111648

**Published:** 2022-11-07

**Authors:** Shu-Dong Zhang, Li-Zhen Ling

**Affiliations:** School of Biological Science and Technology, Liupanshui Normal University, Liupanshui 553004, China

**Keywords:** *Sorbus* sensu stricto, plastome, phylogenetic analysis

## Abstract

*Sorbus* L. is a genus of controversy on its taxonomic concept due to great variations in morphological characteristics. *Sorbus* sensu stricto species, being monophyletic, are characterized as pinnate leaves. However, phylogenetic relationships of these species are somewhat fluid based on morphological characteristics and genetic markers. In this study, the chloroplast (cp) genomes of eight *Sorbus* s. str. species were characterized and compared with those of twelve released species in this genus. Our results indicated that the plastomes of the twenty *Sorbus* species had a conserved quadripartite structure, and 129 annotated genes had the same order and showed a good collinearity. Additionally, numerous SSRs were observed in the cp genome of each *Sorbus* species; most of the sequence variations appeared in non-coding regions, and four intergenic regions were identified as mutation spots. By contrast, protein-coding genes showed low variations under purifying selection. The phylogenetic and molecular-dating analysis showed that *Sorbus* was resolved into two major clades, in which *S. americana* forms one clade originating at 51.78 Ma, and the rest of *Sorbus* formed another clade, splitting at 36.77 Ma into two sister groups with high support values. These results provide a basis for further studying the phylogenetic relationship and biogeography of *Sorbus* species.

## 1. Introduction

*Sorbus* L. is a deciduous plant that belongs to Amygdaloideae of Rosaceae, which is widespread in the northern temperate regions [1,2,3]. China is regarded as the center of distribution and differentiation of this species, because more than half of the species in this genus have been recorded in China [4]. This genus is famous for its ornamental values, which can exhibit various leaf and fruit colors in different seasons [5]. In addition, the edible fruits (berries) are rich in nutritional materials, such as vitamins, polysaccharides, organic acids, and minerals, which are usually used raw in processing into preserves and beverages [6]. Meanwhile, numbers of studies have reported that the fruits of this genus are appreciated for their medicinal properties. It has so far been indicated that chemical constituents of the genus *Sorbus* include phenolic compounds, triterpenes, sterols, carboxylic acids, coumarins, and cyanogenic glycosides, which have antidiabetic, anti-inflammatory, antimicrobial, and antioxidant effects [6]. With more findings of the values of this genus species, some other application potentials have been found, such as insect and disease resistance [5].

The classification of *Sorbus* has been a controversy for a long time. *Sorbus* sensu lato are characterized by both the simple-leaved species and pinnate-leaved species. In 1974, Yü and Lu [4] divided *Sorbus* s. l. into three sections (*Aria*, *Micromeles,* and *Sorbus*), mainly based on the serrated position on leaves and the pairs of leaflets. Subsequently, Phipps et al. (1990) suggested that *Sorbus* s. l. can be divided into six subgenus (*Chamaemespilus* Medikus, *Torminalis* Medikus, *Aria* (Pers.) Host, *Micromeles* Decaisne, *Cormus* Spach, and *Sorbus* sensu stricto) and includes over 250 species [3]. The former four subgenera comprise the simple-leaved species, whereas the latter two subgenera include the pinnate-leaved species. However, several lines of evidence have indicated that *Sorbus* s. l. is polyphyletic, based on the molecular data and morphological characteristics [7,8,9,10]. Therefore, some taxonomists have thought that the genus *Sorbus* should be narrowed in the scope of classification and only include *Sorbus* sensu stricto.

The *Sorbus* sensu stricto (88 species) have high similarities in many morphological characteristics, such as flower structure and color, fruit color and, the numbers of leaflet pairs [11]. The findings have confirmed that *Sorbus* s. str. is a monophyletic group and is classified into two major lineages (*core Sorbus* and *Albo-carmesinae*) [12]. However, a phylogenetic analysis based on a few plastid and nuclear gene fragments found that the relationships and definitions of clades within *core Sorbus* and *Albo-carmesinae* lineages are not well-supported or not consistent with traditional taxonomy based on the morphological characters [12].

In recent decades, the chloroplast (cp) genome has been widely utilized as a phylogenetic marker for investigating plant evolution due to its many advantages. First, the cp genome is small in size, and usually used in comparative studies. In addition, the evolutionary rate of the overall cp genome is slower than that of the nucleotide genome, and the different parts in the cp genome show different rates of nucleotide change, which is appropriate for different level comparisons [13]. Finally, the uniparental inheritance pattern and lack of recombination in the cp genome can simplify analysis. Recently, we analyzed the phylogenetic relationship of Rosaceae based on the complete cp genome to reveal the temporal diversification [7]. In this study, the cp genomes of eight *Sorbus* s. str. species were sequenced using the modern next-generation sequencing approach, and these were compared with those of twelve released species in this genus. We mainly focused on cp genome features, repeat sequences, genome structure rearrangement, sequence variations, and selective pressure, as well as phylogenetic and molecular-dating analysis. This study will further detail the cp genomes of *Sorbus* and provide the basis for studying the phylogenetic relationships within *Sorbus*.

## 2. Materials and Methods

### 2.1. Plant Material and DNA Extraction

Eight *Sorbus* s. str. species, including *S. amabilis*, *S. discolor*, *S. filipes*, *S. hupehensis* var. *hupehensis*, *S. multijuga*, *S. pohuashanensis*, *S. reducta*, and *S. wilsoniana,* were collected from several provinces of China, and the voucher specimens of these species were deposited in Herbarium, Kunming Institute of Botany, CAS (KUN). Detailed information on the samples is shown in Table S1. The total DNA of each sample was isolated from the silica gel-dried leaves by modified CTAB method [14], and a NEB Next Ultra DNA Library Prep Kit for Illumina (New England Biolabs, Ipswich, MA, USA) was used for preparing libraries, which were sequenced on the Illumina HiSeq X-ten. In this study, twelve released *Sorbus* species were also used for a comparative analysis, and the detailed information is shown in Table S1.

### 2.2. Cp Genome Assembly, Annotation, and Comparative Analysis

After the cp genome was de novo assembled using SPAdes software [15], the genes, including protein-coding genes (PCGs), transfer RNAs (tRNAs), and ribosomal RNAs, (rRNAs) were annotated by PGA software [16] with manual adjustments. The annotated cp genome map of *Sorbus* was constructed using the OGDRAW program [17]. The GC content and length of each part of the cp genome were analyzed for 20 *Sorbus* species (8 newly sequenced and 12 available *Sorbus* species) using Geneious software [18]. The plastid sequences from 20 *Sorbus* species were aligned with MAFFT v.6.833 [19] using the default settings. Cp genome sequence homology and collinearity were analyzed using the Mauve software [20]. Pairwise alignments among 20 *Sorbus* cp genomes were performed in the mVISTA program [21], using *S. amabilis* as reference sequence to obtain the overall similarity of cp genomes within *Sorbus* species. The boundary information of the junction of IR, LSC, and SSC was analyzed using the online IRscope tool (https://irscope.shinyapps.io/irapp/, accessed on 24 October 2022).).

The online REPuter software [22] was used to identify four different repeat types: forward, palindrome, reverse, and complement repeats. Additionally, we detected the distribution of microsatellite (simple sequence repeats, SSRs) using MISA web software [23]. In our analysis, we set the threshold of ten, five, four, three, three, and three repeat units for each mono-, di-, tri-, tetra-, penta-, and hexa-nucleotide SSR, respectively. The minimum distance between two SSRs was set to 100 bp.

The nucleotide diversity (pi) values were calculated using a sliding window for cp genomes among the *Sorbus* species with the DNASP 5.0 program [24]. The window length and step size were set to 600 and 200 bp, respectively. The ratio of synonymous (Ks) and non-synonymous (Ka) substitution rates was designed to measure selection pressure on amino acid substitutions [25]. In this study, we extracted the same individual CDS sequence of PCGs and aligned them separately using MAFFT v.6.833 [19]. Then, the alignments were used to calculate the Ka/Ks values, which were lower than, equal to, or greater than 1, indicating purifying selection, neutral evolution, and positive selection, respectively.

### 2.3. Phylogenetic Analysis and Divergence Time Estimation

A total of twenty species were used for the phylogenetic reconstruction of *Sorbus*. Of these, eight species were newly sequenced in this study, and twelve species were downloaded from the NCBI GenBank database (Table S1). In addition, five *Pyrus* species (*P. medvedevii*, *P. oxyprion*, *P. hopeiensis*, *P. pyrifolia,* and *P. pashia*) were used as outgroups. All complete cp genomes were aligned using MAFFT v.6.833 [19] and then separately used to perform the phylogenetic analysis with the Bayesian inference (BI) and maximum likelihood (ML) methods, as previously described [7].

During the calculation of the divergence time, three fossil-based calibration points and one secondary calibration point (lognormal prior distribution) were selected from the literature. (1) Based on the result of Lo and Donoghue [9], the crown node age of *Pyrus* and *Sorbus* was constrained to 45 Ma (A, offset: 45 Ma). (2) The crown of *Sorbus* was dated based on the record of the oldest *Sorbus*-like macrofossils [26] (B, offset: 51 Ma). (3) The stem of clade II-2 (C, offset: 30 Ma) was set to 30 Ma based on the fossil *S.* cf. *tianschanica* from the northwest of China [27]. (4) According to the suggestions of Korotkova et al. [28], the crown node age of *Pyrus* was constrained to 25 Ma (D, offset: 25 Ma).

The node ages of the major clades of *Sorbus* were analyzed using the GTR + GAMMA model in BEAST v.1.8.4 [29]. The divergence time was estimated using a Yule speciation prior and an uncorrelated lognormal model of rate change with a relaxed clock. Two independent runs, each with 20,000,000 generations, were carried out, with sampling every 1000 generations. The effective sample sizes (ESSs) of all parameters were determined (>200) using Tracer v1.6 [30], and the first 25% of the samples was discarded as burn-in. TreeAnnotator v1.8.0 [29] was used to produce a maximum clade credibility chronogram showing the mean divergence time estimates with 95% highest posterior density (HPD) intervals.

## 3. Results and Discussion

### 3.1. Comparative Analysis of the Features and Structure of Cp Genomes in Sorbus

**Figure 1:**
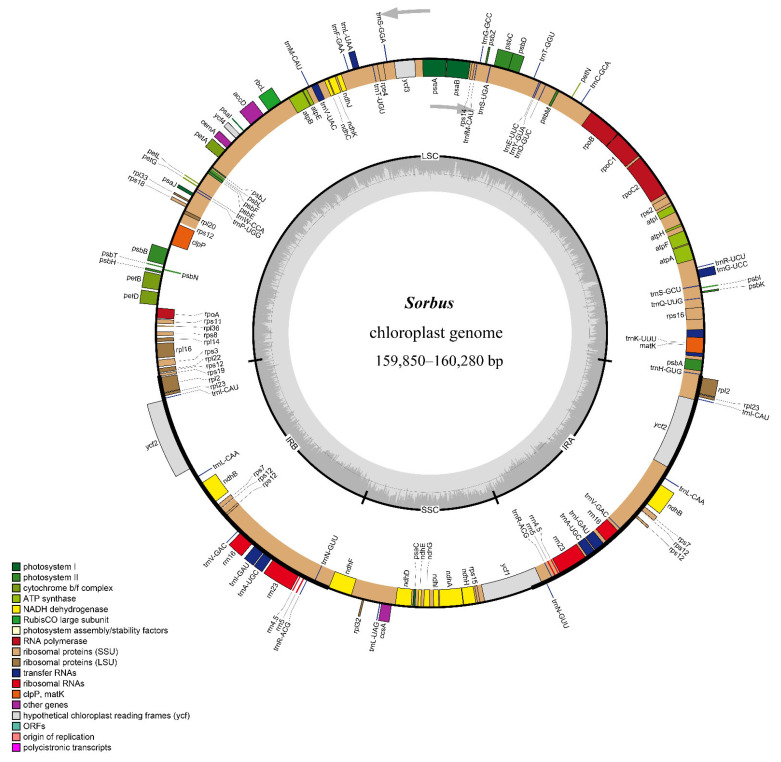
Chloroplast genome map of *Sorbus*. The inner grey circle indicates the GC content of each genome position. Genes in the inner circle of the genomic map are transcribed clockwise and vice versa.

In this study, sequencing of eight different *Sorbus* s. str. species showed an identical cp genome structure. At first, all eight cp genomes exhibited a circular and quadripartite DNA structure with two copies of an inverted repeat (IR) separating two single copy regions, namely, the large and small single copy regions (LSC and SSC, respectively) (Figure 1). This cp genome structure is typical in most land plants [6,13,31] and also found in twelve other publicly available *Sorbus* species (Figure 1). The eight cp genome sizes ranged from 159,850 bp to 160,280 bp, and GC contents rarely changed (36.52–36.58%) (Table 1).

Among four regions, the length of IR regions was nearly identical in newly sequenced and released *Sorbus* species, ranging from 26,314 bp to 26,410 bp (Table 1). The LSC region had the largest length, with 87,803 bp of *S. commixta* to 88,180 bp of *S. pohuashanensis* (Table 1). By contrast, the SSC region had the smallest length, with 19,218 bp of *S. koehneana* to 19,506 bp of *S. aucuparia*.

A total of 129 genes were annotated in each *Sorbus* species, and 17 genes were duplicated in the IR region (Table S2). The majority of these genes were protein-coding genes (PCGs, 84), mostly involved in photosynthesis (Table 1 and Table S2). The remaining genes were transfer RNA (tRNA, 37) and ribosomal RNA (rRNA, 8) genes, and the detailed information is shown in Table S2. In some other genera of Rosaceae (i.e., *Rosa*), the same gene number was found in the same category of gene [32]. In addition, a total of 19 introns occurred in 17 genes among 20 cp genomes (Table S2). Of these, 15 genes had one intron, and two genes had two introns (Table S2). All the genes in 20 cp genomes were organized in an identical order, although these gene transcripts were subjected to cis- or trans-splicing (Figure 1).

**Figure 2:**
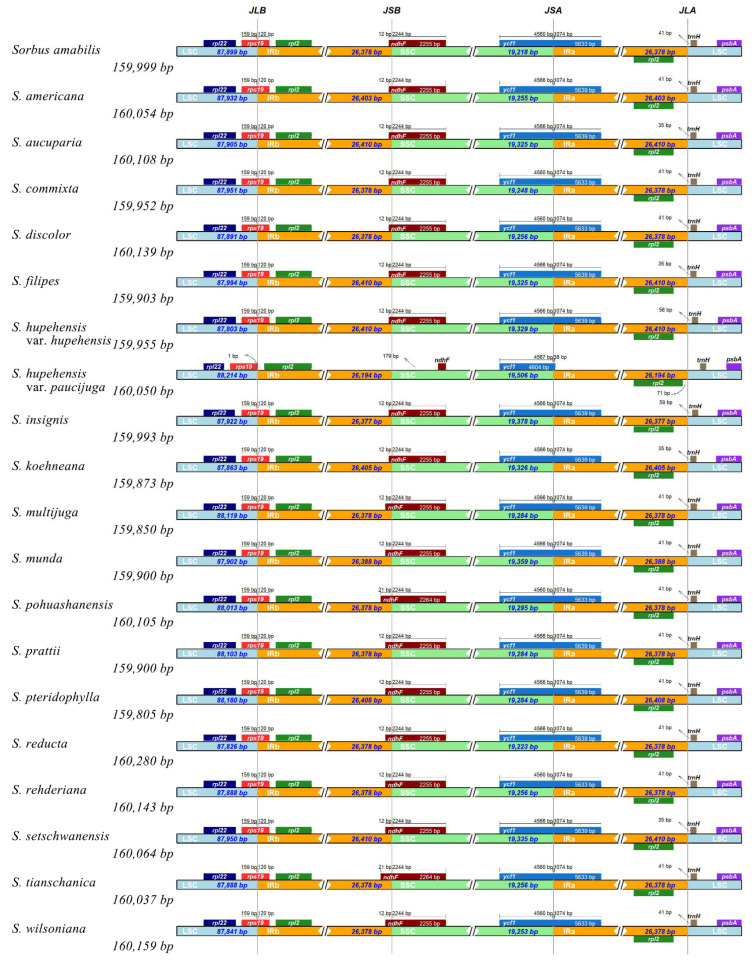
Comparison of the border regions of four chloroplast genome parts among *Sorbus* species.

It has been suggested that the presence of IR is correlated with the stability of gene order [33,34]. For instance, changes of gene order usually co-occur with the loss of the IR region in legumes [35] and conifer [36]. In this study, the IR/SC region boundaries were analyzed and varied slightly across *Sorbus* species. Among *Sorbus* species, the boundaries of IRb/SC were relatively conserved, and two genes (*rps19* and *ndhF*) were detected across the LSC/IRb and SSC/IRb, respectively (Figure 2). There was one exception that a little contraction occurred in the IRb region of the released *S. aucuparia* (Figure 2). The LSC/IRb (JLB) boundary was in the intergenic spacer (IGS) between *rps19* and *rpsl2*, whereas the SSC/IRb (JSB) boundary was flanked *ndhF* with a length of 179 bp away from the 5′ end of this gene (Figure 2). By contrast, the IRa/SC junctions showed quite major differences. The IRa/SSC (JSA) junction was also located within *ycf1*, in which the partial sequence present in the SSC region showed a difference in length (4560–4567 bp) (Figure 2). However, the IRa/LSC (JLA) junctions were located between *rpl2* and *trnH*. The distance between *trnH* or *rpl2* and the boundaries of the JLA exhibited slight changes among the *Sorbus* species, ranging from 35–71 bp (Figure 2). Similarly, the junction of IRa/SC region in *S. aucuparia* showed a difference from other *Sorbus* species (Figure 2). The reason for this sight difference in the individual species needs to be further explored. Therefore, a lack of any change in gene order among the 20 cp genomes of the *Sorbus* species might be correlated with the nearly unchanged IR.

Repeat motifs in plastomes may induce recombination and genome rearrangement [37]. In this study, we analyzed the repeat motifs of four types: palindromic repeat, forward repeat, reverse repeat, and complement repeat. The results revealed that palindromic and forward repeats were the two main repeat motifs across the *Sorbus* cp genomes, ranging from 31–46% for the former and 38–58% for the latter (Figure S1). Although the repeat motifs in each *Sorbus* species varied little, the majority of them were located in non-coding regions within the LSC region (Table S3). To further determine whether the rearrangement occurred in the cp genome of *Sorbus* species, we conducted multiple genome alignments and found that there were no changes in gene order or orientation in cp genomes across the 20 *Sorbus* species (Figure S2). These analyses demonstrate that no genes were disrupted by the repeats, and no genome rearrangement occurred over vast periods of evolutionary time in *Sorbus*. Altogether, the results indicated that eight sequenced cp genomes shared an identical gene structure (gene order and organization) with twelve released *Sorbus* species (Figure 1 and Figure S2), suggesting evolutionary conservativism in *Sorbus*.

### 3.2. Sequence Polymorphism Variation and Divergence Analysis in Sorbus

**Figure 3:**
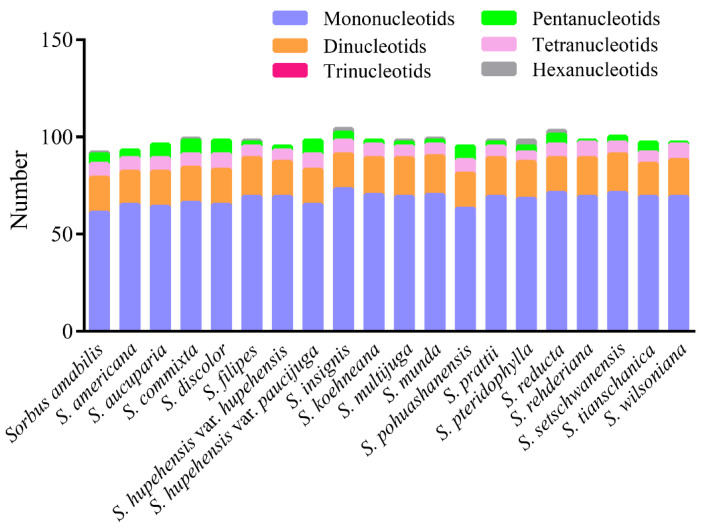
Number of six SSR types examined in the 20 *Sorbus* chloroplast genomes.

Simple sequence repeats (SSRs) have a high degree of polymorphism and are usually employed as the molecular marker for species identification [38] and phylogenetic analyses [39]. SSRs contain tandem repeats of 1–6 bp and are widely distributed in the plastomes in plants [40]. In this study, we analyzed the mono-, di-, tri-, tetra-, penta-, and hexa-nucleotide repeat units, and a total of 1954 SSRs were identified in the 20 *Sorbus* species (Figure 3). Among them, mononucleotide repeats were the most abundant repeat motif, followed by dinucleotide repeats in each *Sorbus* species (Figure 3). We also found that there were more tetranucleotide repeats than pentanucleotide repeats in most of the *Sorbus* species. Hexanucleotide repeats were very rare across these cp genomes and were not even found in some species (Figure 3). However, trinucleotide repeats were not identified in all 20 *Sorbus* species (Figure 3). Moreover, mononucleotide and dinucleotide repeats showed a certain base preference. A/T showed the highest single nucleotide repeats, whereas the majority of dinucleotide repeats were AT/AT repeats (Table S4). These results are consistent with A/T enrichment in the cp genomes of plants [40].

**Figure 4:**
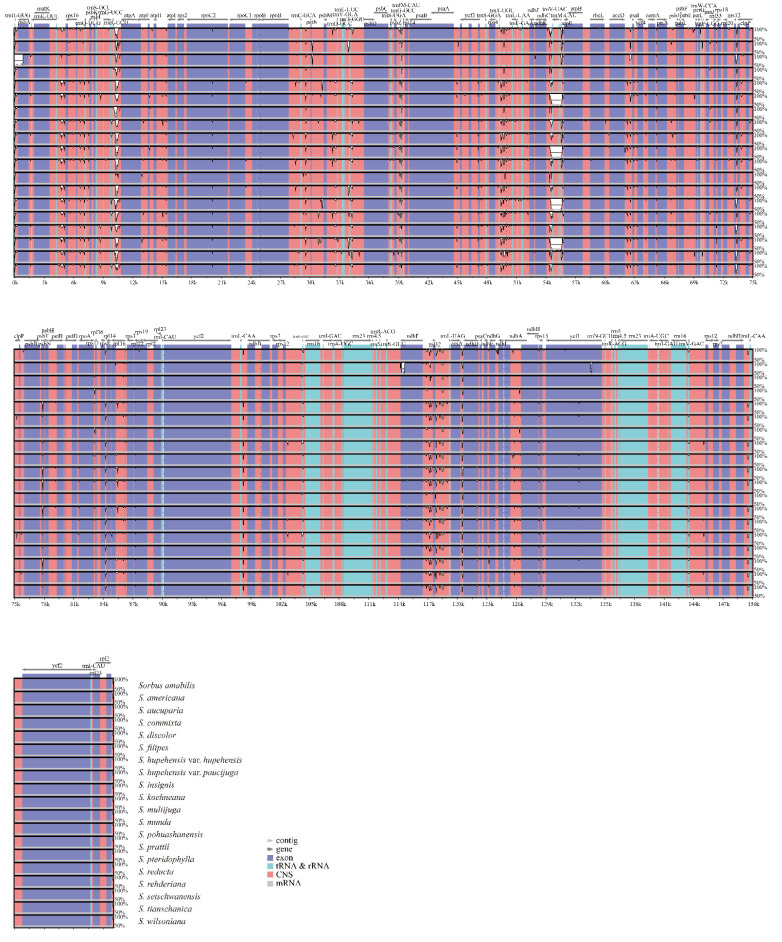
Alignment of 20 *Sorbus* chloroplast genomes using mVISTA.

**Figure 5:**
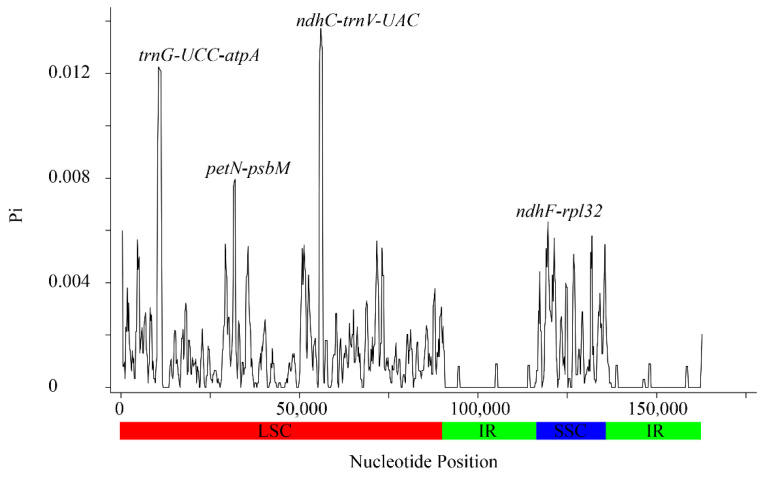
Sliding window analysis of pi values among 20 *Sorbus* species. X-axis, position of the midpoint of a window; Y-axis, nucleotide diversity of each window. (Window length: 600 bp, step size: 200 bp).

In addition, the plastomes of the 20 *Sorbus* species were compared and plotted with mVSITA, using the cp genome of *S. amabilis* as a reference. Our results indicated that the complete cp genomes among the *Sorbus* species were relatively conserved, and two IR regions were the most conserved, whereas the SC regions (especially the LSC) were more divergent and variable (Figure 4). These variations were mainly distributed in non-coding regions, whereas most of the protein-coding regions had a high degree of conservation (Figure 4). Unsurprisingly, these findings were consistent with the patterns observed in many other plants [41,42]. Sliding window analysis further revealed highly variable regions in the plastomes of the 20 *Sorbus* species. The nucleotide diversity (pi) was calculated, and we found that the genomic regions of LSC and SSC had more nucleotide diversity than the two repeat regions (IRa and IRb) (Figure 5), which was consistent with the mVISTA results. In addition, four variable regions with pi values higher than 0.06 were detected, including *trnG-UCC-atpA*, *petN-psbM*, *ndhC-trnV-UAC,* and *ndhF-rpl32* (Figure 5). Of these, *trnG-UCC-atpA* and *ndhC-trnV-UAC* showed the highest diversity, and both the pi values were greater than 0.12, which might have potential value for inferring the phylogenetic evolutionary relationship of the *Sorbus* species. Based on these results, the non-coding regions underwent more variations and divergences than the protein-coding regions. Subsequently, we further calculated the ratio of nonsynonymous (Ka) and synonymous (Ks) substitutions on 79 unique PCGs. The results indicated that the Ka/Ks values of all genes in these *Sorbus* species were less than one, suggesting that all the PCGs were under strong purifying selection (Table S5). Meanwhile, these results demonstrate that these genes might be very important in their functions during the evolution of the *Sorbus* species, and less variations occurred.

### 3.3. Phylogenetic Analysis of Sorbus

**Figure 6:**
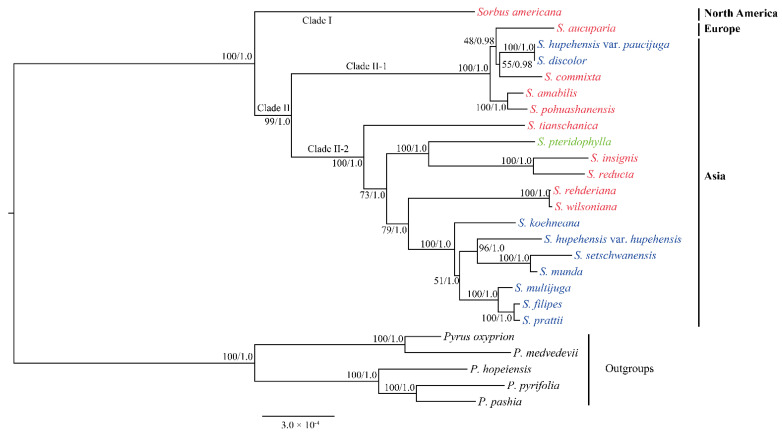
The phylogenetic tree of 20 *Sorbus* species based on chloroplast genome sequences. The species’ names marked with red, blue, and green indicate that the species has red or orange-red, pink, or white fruit, respectively. Numbers at nodes correspond to maximum likelihood bootstrap percentage and the posterior probability of Bayesian inference.

Chloroplast genomes have been characterized for reconstructing phylogenies of plants using three different approaches: restriction fragment/site comparisons, structural rearrangements, and sequence variations [13]. In the present study, the cp genomes of eight *Sorbus* species were newly sequenced and compared with those of twelve released species in this genus. The comparative results indicated that the overall structure of the cp genomes of 20 *Sorbus* species was highly conserved (Figure 1 and Figure S2). Genetic variations (i.e., SSR) were observed in the cp genomes (Figure S2, Figure 4 and Figure 5) and can thus be used for reconstructing the phylogeny of *Sorbus*. ML and BI analysis results showed that the tested *Sorbus* species formed an identical tree topology (Figure 6). We found that *S. americana* from North America was the basal species in *Sorbus,* with 100% bootstrap support and 1.0 posterior probability (Clade I). The remaining 19 *Sorbus* species were classified into two sister groups (Clade II-1 and Clade II-2) (Figure 6). One sister group (Clade II-1) contained six *Sorbus* species: five species from Asia (*S. discolor*, *S. hupehensis* var. *paucijuga*, *S. pohuashanensis*, *Sorbus amabilis*, *S. commixta*) and *S. aucuparia* from Europe. The other 13 *Sorbus* species from Asia formed another sister group (Clade II-2), in which *S. tianschanica* was an early divergent species. All these major clades were strongly supported with high bootstrap support and posterior probability values (Figure 6).

In two previous studies [9,12], phylogenetic analyses resolved two major lineages within *Sorbus* based on nucleotide and chloroplast segments. However, the phylogenetic trees indicated that *S. americana* from North America was embedded into one clade with *S. aucuparia* from Europe and some *Sorbus* species from Asia [9,12]. These topological relationships within *Sorbus* apparently did not coincide with our results (Figure 6). Further, the phylogenetic results of the two studies have a lower support resolution of clades, and the phylogenetic relationships between *Sorbus* and other genera or infrageneric phylogeny of *Sorbus* conflicted [9,12]. Li et al. [12] attributed their conflict to the selection of different genetic markers, whereby four nucleotide segments (*LEAFY-2*, *GBSSI-1*, *SBEI* and *WD*) and one chloroplast segment (*rps16-trnK*) were used in his study. By contrast, another study used a greater number of chloroplast markers (*trnL-trnF*, *trnK + matK*, *rpl16* intron, *rps16* intron, *atpB-rbcL*, *rbcL*, *trnG-trnS*, *rpl20-rps12*, *trnC-ycf6*, *psbA-trnH*, and *trnH-rpl2*) and one nucleotide segment (*ITS*) [9]. These different topologies exert an important influence on the dispersal route of *Sorbus*. Therefore, we will collect more *Sorbus* species from different geographic origins to further determine their topological relationship.

In addition, fruit color is usually used as an indicator of traditional taxonomy within *Sorbus* [4]. In our results, the species in Clade I and II-1 were mainly characterized by red or orange-red fruits, whereas most species of Clade II-2 had white fruits (Figure 6). However, few of the species with different fruit colors were nested in the two sister groups of Clade II, such as two species with white fruit: *S. discolor* and *S. hupehensis* var. *paucijuga* in Clade II-1. Of course, there was low nodal support in infrageneric phylogeny in our results. For example, a low support value (< 50%) occurred between *S. aucuparia* from Europe and three other Asian species (Figure 6). These phenomena also appeared in the phylogenetic results of two previous studies [9,12]. Apomixis, hybridization, and polyploidy are common in *Sorbus* [43,44]. Therefore, infrageneric classification of *Sorbus* becomes more complex, and the fruit color alone cannot be used as a diagnostic characteristic for the clades within *Sorbus*.

**Figure 7:**
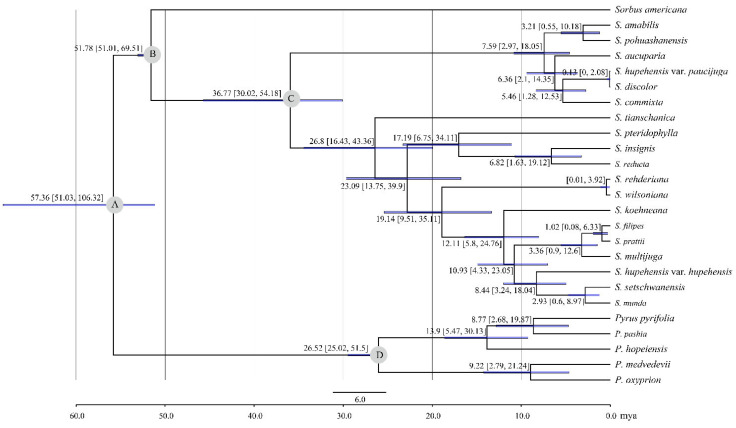
Divergence time of 20 *Sorbus* species derived from BEAST. A, B, C, and D with gray circle represent calibration points. Blue bars represent 95% highest posterior density.

The divergence time between *Sorbus* and *Pyrus* was estimated at 57.36 Ma in the Paleocene, with a 95% HPD range of 51.03–106.32 Ma (Figure 7). The basal node for *S. americana* (Clade I) showed a divergence time of 51.78 Ma (51.01–69.51 Ma, 95% HPD) (Figure 7). These results did not coincide with a previous study, in which *Sorbus* diverged from *Osteomeles* and *Dichotomanthes* at about 31 Ma, and the split within *Sorbus* occurred at about 41 Ma [12]. It has been reported that the oldest *Sorbus*-like macrofossils from North America might date back to 50–52 Ma [45]. Apparently, the divergence time of the *Sorbus* genus or the species from North America was inconsistent with the fossil record in this previous study [12]. Moreover, Clade II-1 and II-2 were split at 36.77 Ma (30.02–54.18 Ma, 95% HPD) in the Eocene (Figure 7). *Sorbus tianschanica* within Clade II-2 originated at 26.8 Ma (16.43–43.36 Ma, 95% HDP) during the late Eocene to Oligocene (Figure 7). The origination of the remainders of *Sorbus* within Clade II occurred in the Miocene (Figure 7), which is similar to the report of Li et al. [12].

## 4. Conclusions

In this study, the cp genomes of eight *Sorbus* s. str. species were newly sequenced and comparatively analyzed with twelve released species in this genus. Our results indicated that all cp genomes of the *Sorbus* species had a typical quadripartite DNA structure and showed little genome size variation, ranging from 159,850 bp to 160,280 bp, and a stable GC level (36.52–36.58%). All cp genomes of *Sorbus* contained the same gene content, indicating no gene loss or intron loss occurred during evolution. Moreover, these genes were organized in the same order and had a good collinearity among all cp genomes of the *Sorbus* species, although long repeat units were found in each species. Therefore, these results indicate that no cp genome of the *Sorbus* species had a rearrangement in structure. Comparative analyses demonstrated that numerous SSRs were observed in the cp genome of each *Sorbus* species, and most sequence variations appeared in non-coding regions. By contrast, the protein-coding genes were all under purifying selection, and four intergenic regions were identified as mutation spots. The phylogenetic analysis based on the complete cp genomes showed that *Sorbus* was resolved into two major clades, in which *S. americana* from North America formed Clade I and originated at 51.78 Ma, and the other species from Europe and Asia were divided into two sister groups (Clade II-1 and Clade II-2) with high support values, which were split at 36.77 Ma. These results not only enrich the data on the cp genomes of *Sorbus*, but also provide the basis for further studying the phylogenetic relationship and biogeography of the *Sorbus* species.

## Figures and Tables

**Table 1:** Summary of complete plastomes of *Sorbus* species.

Species	PCG	tRNA	rRNA	Total Number	Length of Plastome (bp)	Length of LSC (bp)	Length of IR (bp)	Length of SSC (bp)	GC Content (%)
*Sorbus amabilis* *	84	37	8	129	159,999	87,863	26,405	19,326	36.55
*S. discolor* *	84	37	8	129	160,139	87,994	26,410	19,325	36.53
*S. filipes* *	84	37	8	129	159,903	87,891	26,378	19,256	36.58
*S. hupehensis* var. *hupehensis* *	84	37	8	129	159,955	87,951	26,378	19,248	36.56
*S. multijuga* *	84	37	8	129	159,850	87,841	26,378	19,253	36.58
*S. pohuashanensis* *	84	37	8	129	160,105	87,950	26,410	19,335	36.54
*S. reducta* *	84	37	8	129	160,280	88,180	26,408	19,284	36.55
*S. wilsoniana* *	84	37	8	129	160,159	88,119	26,378	19,284	36.54
*S. americana* **	84	37	8	129	160,054	87,922	26,377	19,378	36.55
*S. aucuparia* **	84	37	8	129	160,108	87,974	26,314	19,506	36.54
*S. commixta* **	84	37	8	129	159,952	87,803	26,410	19,329	36.55
*S. hupehensis* var. *paucijuga* **	84	37	8	129	160,050	87,905	26,410	19,325	36.55
*S. insignis* **	84	37	8	129	159,993	87,932	26,403	19,255	36.56
*S. koehneana* **	84	37	8	129	159,873	87,899	26,378	19,218	36.58
*S. munda* **	84	37	8	129	159,900	87,888	26,378	19,256	36.57
*S. prattii* **	84	37	8	129	159,900	87,888	26,378	19,256	36.58
*S. pteridophylla* **	84	37	8	129	159,805	87,826	26,378	19,223	36.59
*S. rehderiana* **	84	37	8	129	160,143	88,103	26,378	19,284	36.54
*S. setschwanensis* **	84	37	8	129	160,064	88,013	26,378	19,295	36.54
*S. tianschanica* **	84	37	8	129	160,037	87,902	26,388	19,359	36.55

Note: PCG indicates protein-coding gene. * indicates the newly sequenced *Sorbus* species in this study, and ** indicates the publicly available *Sorbus* species.

## Data Availability

The datasets generated and analyzed during the current study can be accessed in the NCBI GenBank database (https://www.ncbi.nlm.nih.gov/genbank/), and the accession numbers are listed in Table S1.

## Supplementary Materials

The following supporting information can be downloaded at: https://www.mdpi.com/article/10.3390/biom12111648/s1, Figure S1: Number of four repeats in the cp genomes of 20 *Sorbus* species; Figure S2: Collinearity analysis of chloroplast genomes across 20 *Sorbus* species. Local collinear blocks are labeled by the same color; Table S1: The information of 20 *Sorbus* species used in this study; Table S2: Gene information of the different functional categories of 20 *Sorbus* plastomes; Table S3: Details of the repeats in each *Sorbus* species; Table S4: Details of SSRs in the cp genomes of 20 *Sorbus*; Table S5: The Ka/Ks ratios of protein-coding genes.
