# Supplementary material for: Molecular Structure and Phylogenetic Analyses of the Plastomes of Eight Sorbus Sensu Stricto Species
Source: Biomolecules. 2022 Nov 7;12(11):1648. doi: 10.3390/biom12111648 (PMC9687737; doi:10.3390/biom12111648)
Supplement: Supplementary file 1 [file biomolecules-12-01648-s001.zip › Table S1 The information of 20 Sorbus species used in this study.pdf]

Table S1. The information of 20 *Sorbus* species used in this study.

Species	Accession Number	Geographical origin	Voucher number
Sorbus amabilis	OP613262	Anhui	Tangxs0324
S. discolor	OP613260	Shandong	Lilan702
S. filipes	OP613264	Yunnan	YMT3800-1-2003
S. hupehensis var. *hupehensis*	OP613258	Sichuan	Gaoxf-0945
S. multijuga	OP613261	Sichuan	LuoP202
S. pohuashanensis	OP613257	Liaoning	Caow5157
S. reducta	OP613259	Sichuan	LiJ307
S. wilsoniana	OP613263	Sichuan	YangQE3462
S. prattii	MK814479	Asia	
S. setschwanensis	MK914535	Asia	
S. commixta	MK920288	Asia	
S. tianschanica	MK920289	Asia	
S. aucuparia	MT610101	Europe	
S. insignis	MT677871	Asia	
S. munda	MT683851	Asia	
S. hupehensis var. *paucijuga*	MT916771	Asia	
S. koehneana	MW429483	Asia	
S. americana	MZ984219	North America	
S. pteridophylla	MZ984223	Asia	
S. rehderiana	OK012001	Asia	
