# Supplementary material for: Molecular Structure and Phylogenetic Analyses of the Plastomes of Eight Sorbus Sensu Stricto Species
Source: Biomolecules. 2022 Nov 7;12(11):1648. doi: 10.3390/biom12111648 (PMC9687737; doi:10.3390/biom12111648)
Supplement: Supplementary file 1 [file biomolecules-12-01648-s001.zip › Table S2 Gene information of the different functional categories of 20 Sorbus plastomes.pdf]

Table S2. Gene information of the different functional categories of 20 *Sorbus* plastomes

Function of Genes	Group of Genes	Gene name
Photosynthesis	Photosystem I	*psaA*, *psaB*, *psaC*, *psaI*, *psaJ*
	Photosystem II	*psbA*, *psbB*, *psbC*, *psbD*, *psbE*, *psbF*, *psbH*, *psbI*, *psbJ*, *psbK*, *psbL*, *psbM*, *psbN*, *psbT*, *psbZ*
	Cytochrome b/f complex	*petA*, *petB*[a], *petD*[a], *petG*, *petL*, *petN*
	ATP synthase	*atpA*, *atpB*, *atpE*, *atpF*[a], *atpH*, *atpI*
	NADH-dehydrogenase	*ndhA*[a], *ndhB*[*a], *ndhC*, *ndhD*, *ndhE*, *ndhF*, *ndhG*, *ndhH*, *ndhI*, *ndhJ*, *ndhK*
	Large subunit Rubisco	*rbcL*
Protein synthesis and DNA-replication	Subunits of RNA polymerase	*rpoA*, *rpoB*, *rpoC1*[a], *rpoC2*
	Ribosomal protein small subunit	*rps2*, *rps3*, *rps4*, *rps7*[*], *rps8*, *rps11*, *rps12*[*b], *rps14*, *rps15*, *rps16*[a], *rps18*, *rps19*
	Ribosomal protein large subunit	*rpl2*[*a], *rpl14*, *rpl16*[a], *rpl20*, *rpl22*, *rpl23*[*], *rpl32*, *rpl33*, *rpl36*
	Transfer RNAs	*trnA-UGC*[* a], *trnC-GCA*, *trnD-GUC*, *trnE-UUC*, *trnF-GAA*, *trnfM-CAU*, *trnG-GCC*, *trnG-UCC*[a], *trnH-GUG*, *trnI-CAU*[*], *trnI-GAU*[* a], *trnK-UUU*[a], *trnL-CAA*[*], *trnL-UAA*[a], *trnL-UAG*, *trnM-CAU*, *trnN-GUU*[*], *trnP-UGG*, *trnQ-UUG*, *trnR-ACG*[*], *trnR-UCU*, *trnS-GCU*, *trnS-GGA*, *trnS-UGA*, *trnT-GGU*, *trnT-UGU*, *trnV-GAC*[*], *trnV-UAC*[a], *trnW-CCA*, *trnY-GUA*
	Ribosomal RNAs	*rrn4.5*[*], *rrn5*[*], *rrn16*[*], *rrn23*[*]
Other genes	Maturase	*matK*
	Translation initiation factor	*infA*
	C-type cytochrome synthesis gene	*ccsA*
	Acetyl-CoA-carboxylase	*accD*
	Inner membrane protein	*cemA*
	ATP-dependent protease	*clpP*[b]
Genes of unknown function	Conserved hypothetical gene	*ycf1*, *ycf2*[*], *ycf3*[b], *ycf4*

Note: * indicates the duplicated gene number of repeat units is 2; [a]Gene contains a single intron; [b]Gene contains two introns
