# Supplementary figures and images for: Molecular Structure and Phylogenetic Analyses of the Plastomes of Eight Sorbus Sensu Stricto Species
Source: Biomolecules. 2022 Nov 7;12(11):1648. doi: 10.3390/biom12111648 (PMC9687737; doi:10.3390/biom12111648)

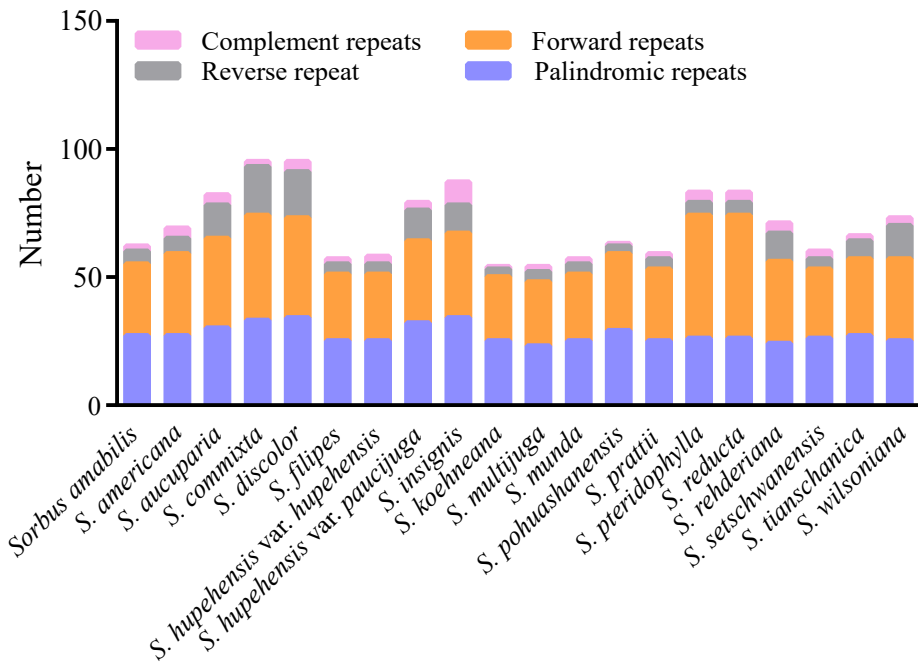

Supplement: Supplementary file 1 [file biomolecules-12-01648-s001.zip › Figure S1 Number of four repeats in the cp genomes of 20 Sorbus species.pdf]

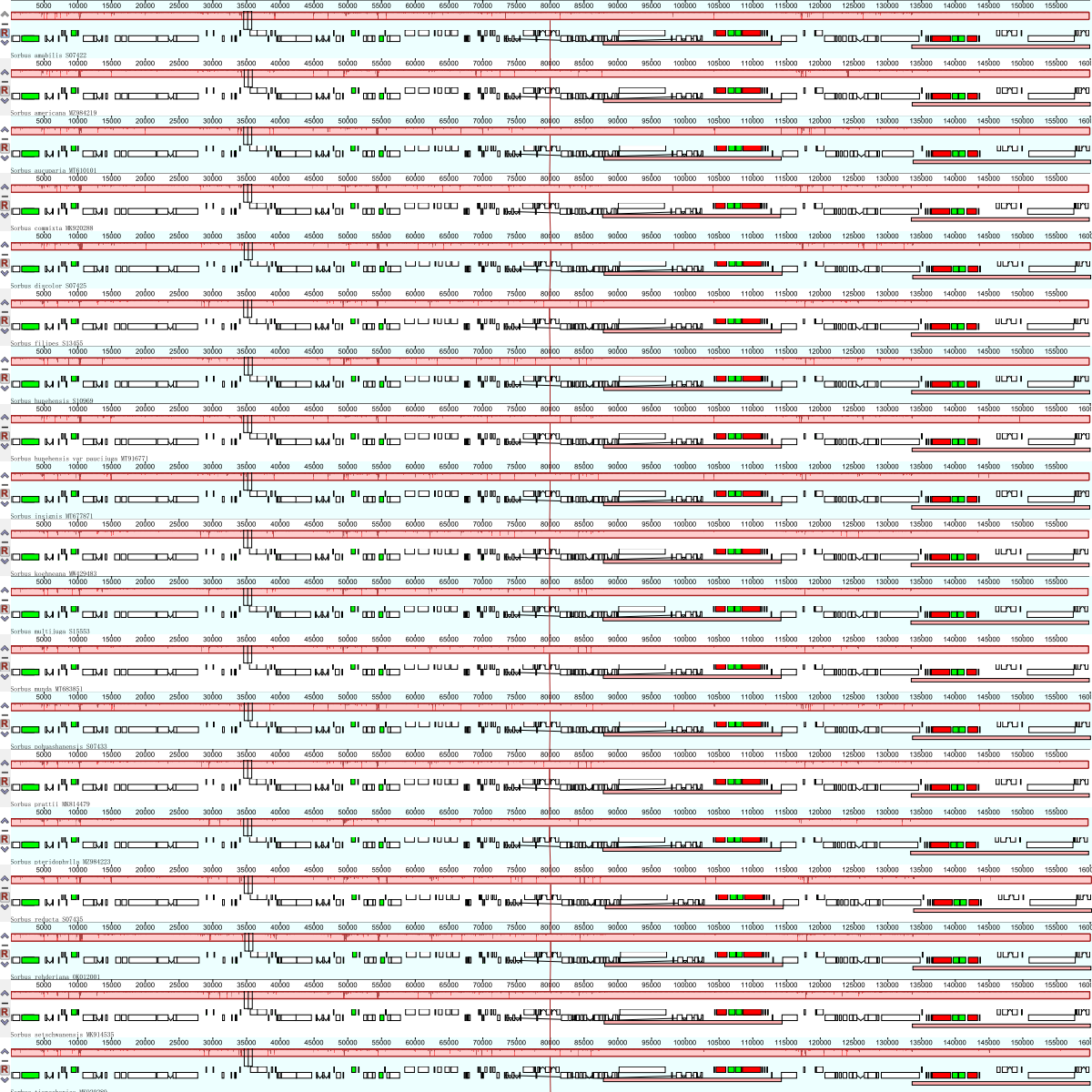

Supplement: Supplementary file 1 [file biomolecules-12-01648-s001.zip › Figure S2 Collinearity analysis of chloroplast genomes across 20 Sorbus species.pdf]
